# Distinguishing between sea turtle foraging areas using stable isotopes from commensal barnacle shells

**DOI:** 10.1038/s41598-019-42983-4

**Published:** 2019-04-25

**Authors:** Ryan M. Pearson, Jason P. van de Merwe, Michael K. Gagan, Colin J. Limpus, Rod M. Connolly

**Affiliations:** 1 Australian Rivers Institute - Coasts & Estuaries, and School of Environment & Science, Griffith University, Gold Coast, Queensland 4222 Australia; 2 Research School of Earth Sciences, Australian National University, Canberra, Australian Capital Territory 2600 Australia; 3 School of Earth and Environmental Sciences, The University of Queensland, Brisbane, Queensland 4072 Australia; 4 Threatened Species Unit, Department of Environment and Science, Brisbane, Queensland 4102 Australia

**Keywords:** Animal migration, Conservation biology, Population dynamics, Stable isotope analysis

## Abstract

Understanding the movement behaviour of marine megafauna within and between habitats is valuable for informing conservation management, particularly for threatened species. Stable isotope analyses of soft-tissues have been used to understand these parameters in sea turtles, usually relying on concurrent satellite telemetry at high cost. Barnacles that grow on sea turtles have been shown to offer a source of isotopic history that reflects the temperature and salinity of the water in which the host animal has been. We used a novel method that combines barnacle growth rates and stable isotope analysis of barnacle shells (δ^18^O and δ^13^C) as predictors of home area for foraging sea turtles. We showed high success rates in assigning turtles to foraging areas in Queensland, Australia, based on isotope ratios from the shells of the barnacles that were attached to them (86–94% when areas were separated by >400 km). This method could be used to understand foraging distribution, migration distances and the habitat use of nesting turtles throughout the world, benefiting conservation and management of these threatened species and may be applied to other taxa that carry hitchhiking barnacles through oceans or estuaries.

## Introduction

Understanding the distribution, migratory pathways, and habitat use of marine fauna is valuable to inform management decisions, especially for threatened species. For example, understanding foraging distributions can help to prioritise conservation efforts via protection of key resources, habitat characteristics or improving fishery/bycatch management within critical habitats. Various methods have been employed across many taxa for these purposes, but with varying success and each with their own limitations. For sea turtles, being long-lived, highly migratory and threatened, understanding spatial and temporal distributions within foraging areas is important for management within and beyond jurisdictional boundaries^1^.

Sea turtles are known to use a variety of habitats throughout their long-lives, with juvenile stages in open-ocean and (for many species) sub-adult and adult stages in coastal foraging areas^2^. As adults, an individual will undergo breeding migrations to an area near to where they hatched^3^ which, for green (*Chelonia mydas*) and loggerhead (*Caretta caretta*) turtles, can be >2,600 km distant from their foraging area^4^. Sea turtles show strong fidelity to foraging, mating and nesting areas throughout their adult lives^4,5^. Turtles in some sub-populations will use different areas in winter and summer (e.g.^5,6^) while others remain in the same foraging area regardless of season (e.g. southwest Pacific loggerheads^4^). Similarly, some sub-populations have turtles that forage in pelagic areas (>200 m deep) as adults (e.g. northwest Pacific loggerheads^7^), while for others there is no evidence suggesting adults feed beyond the continental shelf (e.g. southwest Pacific loggerheads^8^).

Considerable progress has been made toward understanding the foraging distribution of nesting turtles for some sub-populations^9^, largely through the use of satellite telemetry^10^ and also often with concurrent stable isotope analysis (SIA) of sea turtle tissues (e.g.^11–13^). For example, the foraging distribution of nesting turtles and how this varies between years has become quite well understood in the north-west Atlantic through a combination of both techniques (e.g.^14^). However, improving the understanding of adult foraging distributions and how they relate to nesting beach choice would greatly benefit management of many sub-populations (e.g.^1,15^).

Satellite telemetry provides high accuracy and some studies have utilised extensive satellite telemetry derived data (e.g.^16–19^ have sampled between 100–400 turtles). However, replication is limited in some regions^20^ potentially due to the high-costs associated with telemetry studies. The capture-recapture method is comparatively low in cost, but also tends to yield low sample sizes in the short-term due to limited recapture success in foraging areas and provides comparatively limited data (e.g. understanding of behaviour between captures is lacking). In SIA, ratios of light to heavy isotopes (which are variants of the same elements that contain different numbers of neutrons) vary predictably (in both organic or inorganic materials). Variations can be driven by changes in diet, trophic level, or chemical properties such as temperature and salinity – depending on the type of material. SIA techniques allow for higher-replication (due to relatively low cost), but generally also lower spatial resolution compared with satellite telemetry. As a result, SIA of metabolically active tissues (e.g. skin, scute, blood, bone) have been used for distinguishing between foraging areas^21^ and understanding resource use for many taxa (e.g.^22–24^), including sea turtles (see^25^ for review).

SIA studies on sea turtles have almost entirely focused on analysis of soft-tissues^25^, in which isotope ratios are affected by diet choices of the individual. This type of study generally combines a relatively high-volume of satellite telemetry (e.g. 50+ turtles) with SIA of soft-tissues to build a reference library (isoscape or calibration data) of isotopic signals (e.g.^12,26^). These reference libraries are then used to assign foraging areas to individuals that were not tracked. Soft-tissue isotope techniques have been effective in identifying foraging areas of nesting turtles in some regions due to their consistent site fidelity, along with the tendency for females to fast throughout migration and the nesting season. This means that isotope ratios in tissues that have slow turnover rates (e.g. skin and red blood cells) are likely to represent those incorporated from prey consumed in the home foraging area. With sufficient difference in food-web signals between foraging areas, it has been possible to differentiate between areas using C and N isotopes from turtle tissues. However, there remain questions over how much effect individual diet choice has on assignment success. For example, loggerhead turtles are known to be diet generalists at the population level, but specialists as individuals^27^. This suggests that within a foraging area, individual diet choices may result in high variation in isotope ratios and lower ability to identify differences between regions. This phenomenon is partly supported by recent findings that foraging areas with higher prey diversity show higher variation in soft-tissue isotope ratios^28^.

A potential alternative or complementary method to these techniques is the analysis of isotope ratios (δ^13^C and δ^18^O) in the calcareous shells of barnacles commensal on sea turtles. Isotope ratios in balanomorph barnacle shells are known to reflect the physicochemical characteristics (temperature and salinity) of the water in which they form^29–32^. The calcitic shells of acorn barnacles are formed sequentially, primarily via precipitation from the water^33^. It is therefore expected that the contribution diet makes to shell isotope ratios is minimal - estimated between 0–25%^34^. Sequential sampling of barnacle shell can therefore provide information about historical changes in water temperature and salinity (e.g.^32^).

Paleotemperature equations, which describe the temperature dependent fractionation of oxygen isotopes (δ^18^O) in carbonates, have been well established across various taxa such as molluscs^35^ and foraminifera^36^. Additional carbonate materials, such as fish otoliths (e.g.^37–39^) and other bones^40^, have also been useful for inferring water temperatures, salinities, and/or tracing movements. The balanomorph barnacle paleotemperature equation described by^29^, allows conversion between δ^18^O ratios in barnacle shells and water temperature where the oxygen isotope ratio of the seawater itself (δ^18^O_seawater_) is known. This equation shows that as water temperature increases, barnacle shell δ^18^O ratios decrease^29^. Despite this understanding, and extensive use of similar techniques in other fields (e.g.^41^), to date only three studies have attempted to use isotope ratios from commensal barnacle shells to understand the habitat preferences and/or movement of the host^30–32^.

Killingley^31^ demonstrated that barnacle (*Cryptolepas rhachianecti*) shell isotopes (δ^18^O) from two California grey whales (*Eschrichtius robustus*) could be used to estimate water temperatures and aligned these estimates with expected δ^18^O ratios along their north to south migration. Killingley and Lutcavage^32^ showed (using six *Chelonibia testudinaria* barnacles from six loggerhead turtles in the northwest Atlantic) that it is possible to identify when the host transitions between estuarine and oceanic waters. Lastly, Detjen, *et al*.^30^ investigated the foraging habitat of green turtles (*Chelonia mydas*) in the central north Pacific using isotope ratios from commensal *Platylepas sp*. barnacle shells. This study (assessing twelve barnacles from four turtles) found that carbon isotopes were not useful for the purpose in this region, but that oxygen isotopes were able to delineate regional movement patterns at very coarse resolution (identified areas were very large - thousands of km^2^). However, some limitations of this study may have inhibited a complete understanding of the method’s viability. Foremost, a very small barnacle species (~4 mm) was used for which there was no understanding of the barnacle’s growth rates, making it impossible to estimate the age of each sample. An understanding of growth rates could have strengthened these analyses by, at minimum, eliminating areas from which the animal could not have possibly travelled during the time represented in the barnacle’s shell.

Thus, understanding the growth rates of the target barnacle species should form a necessary part of any analysis aiming to understand movement through water bodies using barnacle shell chemistry. A robust understanding of how a barnacle species grows makes it possible to assign an age to each section of the barnacle shell, which can then allow direct comparison between dated samples from different barnacles. The most common barnacle species present on sea turtles worldwide is *Chelonibia testudinaria*. This species has been the subject of three studies investigating its growth rate^42–44^. Most recently, Doell, *et al*.^44^ described a non-linear, von Bertalanffy growth model for *C. testudinaria* barnacles growing on nesting loggerhead turtles in eastern Australia. The rate of shell formation is rapid when young and decreases with age/size. Using this non-linear growth rate it is possible to estimate the age of barnacles and assign a date to isotope samples collected from different areas of the shell.

The aim of this study was to validate a method for extracting and dating barnacle shell samples for isotope analysis (δ^13^C and δ^18^O) and test the accuracy of using isotope ratios from dated barnacle shell samples for distinguishing between the foraging locations of host turtles. We expect this method to be applicable for identifying the migratory origin of mating and nesting sea turtles throughout the world.

## Results

### Method reproducibility

The difference in isotope ratios between 20 paired samples (i.e. pairs from the same barnacle across 20 different barnacles, 40 samples in total) was 0.09 ± 0.07‰ for δ^13^C and 0.18 ± 0.11‰ for δ^18^O (mean ± S.D.). The differences are larger than expected based on machine error within the mass spectrometer alone (given by repeat measurements of NBS-19, which were <0.03‰ for both isotopes). Thus, this error is likely due (at least in part) to heterogeneity within the barnacle.

### Isotopic differences between areas

**Figure 1:**
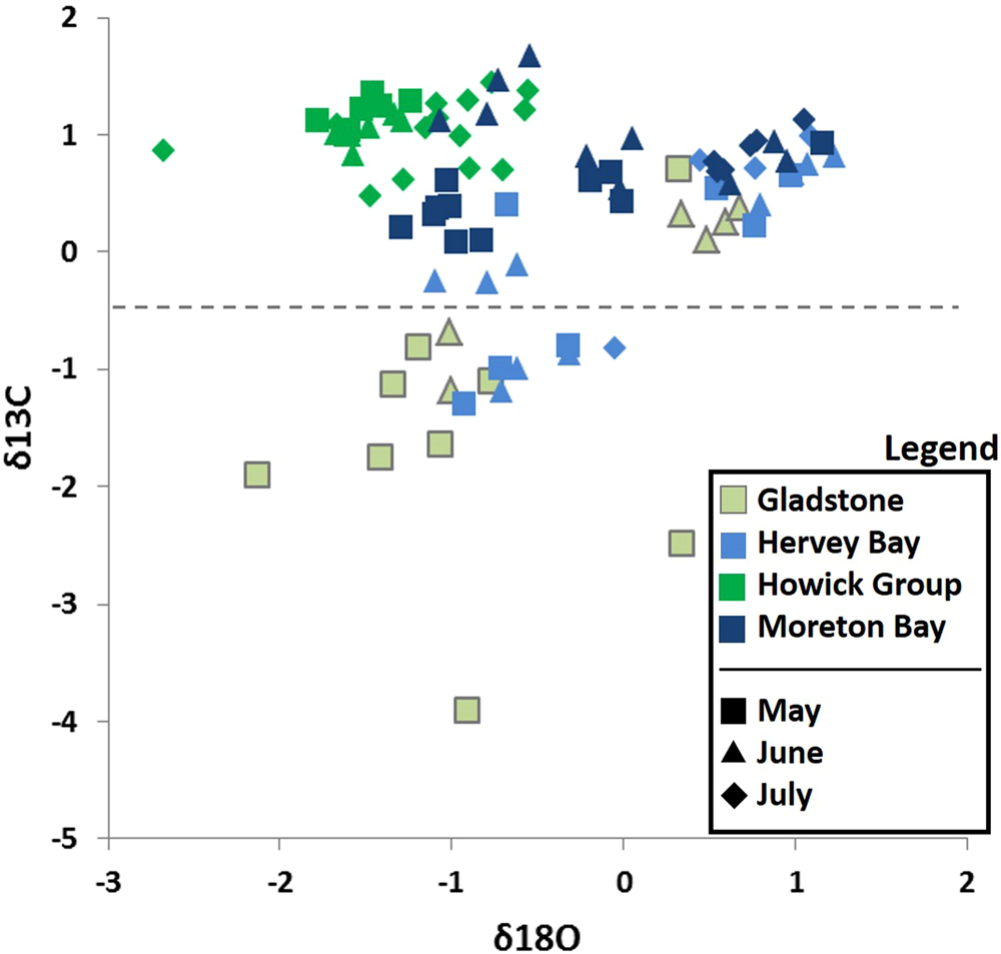
Cross-plot of δ^13^C and δ^18^O (‰) for 93 *Chelonibia testudinaria* barnacle shell samples collected from 27 sea turtles within the four foraging areas. The month in which the shell material was formed is represented by different shapes. The number of turtles and samples within each of the four areas were: Gladstone (7 turtles, 16 samples); Hervey Bay (4 turtles, 23 samples); Moreton Bay (10 turtles, 27 samples), Howick Group (6 turtles, 27 samples).

Ninety-three samples from 27 barnacles (from 27 turtles) were included in these analyses (Fig. 1), except in tests that exclude Hervey Bay samples. The number of turtles and samples within the four areas were: Gladstone (7 turtles, 16 samples); Hervey Bay (4 turtles, 23 samples); Moreton Bay (10 turtles, 27 samples), Howick Group (6 turtles, 27 samples). The number of repetitions within a barnacle ranged from one to seven (mean = 3.4). Comparatively, the largest previous study that assessed turtle movement using barnacle isotopes analysed 70 samples from six barnacles from six turtles^32^ The only other study on sea turtle barnacles assessed a total of twelve barnacles from four turtles, though only nine barnacles from three turtles were included in their analyses due to sample yield being insufficient for mass spectrometry^30^.

MANCOVA returned a significant overall model (p < 0.001 for both isotopes) indicating that there were significant differences the isotope values between areas. The covariate ‘month’ was significant for δ^18^O (p < 0.001), and nearly so for δ^13^C (p = 0.055). Pairwise comparisons show that one or both isotopes are significantly different between each area pairing (Table 1). The distribution of data from Gladstone and Hervey Bay showed signs of bimodality for one or both isotopes, but given the robustness of parametric ANOVA models to mild departures from the normality assumption, as well as the highly significant p values for both isotopes, we consider this test to represent the best available assessment of the data.

**Table 1:** Pairwise (between locations) MANCOVA results for barnacle shell δ^18^O (left/bottom) and δ^13^C (right/top) between the four foraging areas.

		Howick	Gladstone	Hervey	Moreton	
Howick	**δ** ^**18**^ **O**	NA	<0.001*	<0.001*	0.202	**δ** ^**13**^ **C**
Gladstone		<0.001*	NA	<0.001*	<0.001*	
Hervey		<0.001*	0.083	NA	<0.001*	
Moreton		<0.001*	0.377	0.297	NA	

Significant differences are represented by *.

### Assigning location to turtles using barnacle isotope samples

We assessed how management relevant the differences between barnacle isotopes from each areas were via Linear Discriminant Analysis (LDA). This technique quantified how accurate the model was at assigning turtles to their known home area, rather than any of the other three areas tested. The 12 LDA iterations returned overall foraging ground assignment accuracies ranging between 50% and 97% at the sample level, and between 57 and 96% at the turtle level (Table 2; Appendix S1: Table S1). Mean accuracies (from averaging both directions) ranged between 59% and 94% at the turtle level. The Howick Group was the most successful area, with 100% of turtles assigned correctly in all tests. Hervey Bay was the least successful with zero to 75% of turtles being assigned correctly (Table 2; Appendix S1: Table S1).

**Table 2:** Results of Linear Discriminant Analyses using multiple methods to define calibration and validation datasets.

Split method	Areas	Sample Sizes (N)						Assignment success per area (%)								Overall Accuracy (%)		Mean accuracy (%)	
		Turtles			Samples			South						North					
								Gladstone		Hervey		Moreton		Howick					
		Total	Cal	Val	Total	Cal	Val	S	T	S	T	S	T	S	T	S	T	S	T
Alternating samples (AS)	**4**	27	25	26	93	47	46	75	71	45	75	62	78	100	100	71	81	**71**	**78**
	**3**	23	21	22	70	35	35	75	71	NA		86	80	100	100	87	82	**85**	**86**
	**2**	27	25	26	93	47	46	**S** = 94; **T** = 95						100	100	97	96	**97**	**94**
Alternating turtles (AT)	**4**	27	14	13	93	44	49	14	17	0	0	87	90	100	100	50	62	**53**	**59**
	**3**	27	12	11	70	33	37	57	67	NA		93	100	93	100	81	91	**88**	**91**
	**2**	23	14	13	93	44	49	**S** = 94; **T** = 90						100	100	97	92	**97**	**93**

Split method defines how each sample was selected for the calibration or validation datasets. Areas: the number of regions which were used as the grouping variable. Cal: The number of turtles and samples used in the calibration subset and; Val: in the validation subset. Assignment success per area is defined as the number of samples (S) and turtles (T) that were correctly assigned to the areas they were from. Overall Accuracy: the percentage of samples (S) and turtles (T) correctly assigned to their home area across all areas used. Mean Accuracy: the average percentage of samples (S) and turtles (T) that were assigned correctly across both directions. South & North groupings used where only two areas are included. Gladstone, Moreton, and Howick used in three area tests. Only forward iterations shown. Reverse iterations in Appendix I.

Splitting datasets using the AS method (mean 86%) generally provided more accurate assignments than the AT method (mean 81%). Assignment accuracy for both AT and AS methods increased each time an area was removed from the analysis, with assignment success lowest using the AT method and with all four areas included. Both AS and AT methods provided assignment successes >90% when separating between only two areas (North & South; Table 2; Appendix i).

With all four areas in the model (minimum separation approx. 150 km), the Hervey Bay negatively affected overall assignment success and success within each neighbouring area (Gladstone & Moreton Bay). Using the AT method, Hervey Bay returned 0% assignment success, and very low success for either Gladstone (14%) or Moreton Bay (42%) depending on the direction (Table 2; Appendix S1: Table S1). In these tests, Gladstone showed low success in the forward iteration (14%), and high (78%) in the reverse direction, while Moreton Bay showed the opposite trend (90% forward, 40% reverse). The AS method with four areas was more successful (mean = 78%), but remained the lowest of all AS attempts (Table 2; Appendix S1: Table S1).

With only three foraging areas included (minimum separation approx. 400 km), assignment success increased substantially overall (AS = 86%; AT = 91%) and within each of the southern areas (Gladstone mean = 75%, max = 100%; and Moreton Bay mean = 88%, max = 100%) (Table 2; Appendix S1: Table S1).

The best assignment success came when combining the three southern regions into one group (South) and comparing to the Howick group samples (North). This method returned assignment mean successes of 94% (AS) and 93% (AT) at the turtle level, with only one turtle being incorrectly assigned in three out of four tests, and two incorrect in the fourth (reverse AS method). At the sample level both AS and AT methods returned identical 97% accuracies (Table 2; Appendix S1: Table S1).

## Discussion

Here we show that isotope ratios (δ^13^C and δ^18^O) of the calcareous shells from barnacles commensal on sea turtles can be used to distinguish between foraging areas with success varying at different spatial scales. We demonstrate that incorporating barnacle growth rates (to assign dates to samples) strengthens assignment success and allows for comparison to the characteristics of water bodies (e.g. SST) at the time of shell deposition.

In eastern Australia, it is possible to assign turtles to foraging areas with high success, up to 94% assignment accuracy, when separating assignment to northern or southern areas in Queensland, Australia, regions separated by about 1,100 km. We expect the differences between our north and south groups is driven simultaneously by latitudinal gradients in temperature, and salinity gradients between estuarine and marine waters. This spatial scale is comparable to that using soft-tissue methods in the northwest Atlantic where the eastern US coast was separated into four areas, each up to 1,600 km across (e.g.^12^), and in the northeast Pacific where three foraging areas were each separated by approximately 1,000 km^45^. In the Mediterranean, stable isotopes were used to assign nesting turtles to two foraging areas, each separated by about 1,000 km and approximately 600–900 km across^46,47^.

At smaller spatial scales, it was still possible to achieve high assignment success if regions were sufficiently separated. For example, we show that assignment success is still high (up to 91%) when separating between areas with a minimum distance between them of approximately 400 km. However, with all four areas included, the minimum distance between two areas was approximately 150 km. Overall assignment success in this test was lowest (59% of turtles correct), driven by the low assignment success of Hervey Bay turtles, and their effect on the assignment success of adjacent areas. It is possible that higher replication within each region could reduce this confusion and allow for higher assignment success at this scale (as may be suggested by the higher assignment success in the four area AS method), but it is also possible that separation of regions by only 100–200 km is too fine for the method to be effective in this region.

When removing Hervey Bay from the analysis (increasing minimum separation to approx. 400 km), assignment success was considerably higher with 91% of turtles being correctly assigned. Averaging success from the forward and reverse directions shows that success remained high within the two closest areas: Gladstone (83% of turtles) and Moreton Bay (90%). In this test, once again we expect both temperature and salinity to be driving differences, however the effect is likely different between paired areas. Gladstone is distinguishable from the other two areas due to a high estuarine (low salinity) influence, with the temperature effect being small in comparison. The other two areas (Howick and Moreton Bay) appear to have been mostly marine in nature during the period and are therefore likely being separated primarily by temperature differences.

The ability to assign a date to barnacle shell samples based on the work of Doell *et al*.^44^ allowed the significant effect of month on isotope ratios within each region to be used as a predictor in the LDA model. Without this ability, it is likely that assignment success would have been considerably lower in all tests. For example, samples from the Howick Group and Moreton Bay show similar ratios for both isotopes, and appear to occupy the same space when viewed without the effect of month (Fig. 1). However, there is no overlap in values between these areas within a given month, thus allowing the LDA model to better distinguish between the two areas than if this parameter had not been included.

By applying the techniques presented here in future assessments of turtle movement, it is feasible that nesting turtles could be accurately assigned to foraging areas separated by as little as 400 km, especially when there is a variable effect of salinity between different estuaries as seen here. Given the overlap in temperature ranges between the Howick Group and Moreton Bay, it is likely that the resolution in marine habitats in eastern Australia will fall between 400 and 1100 km.

This resolution is finer than has been achieved in most previous geographic isotope studies on sea turtles, but not as fine as has been achieved in some other, less mobile taxa such as scallops and jellyfish^48^. Recently, however, Ceriani, *et al*.^14^ identified foraging hotspots for northwest Atlantic loggerhead turtles, with some of the hotspots covering notably smaller areas (smallest = 4,800 km^2^) than has been achieved in previous isotope studies. This was supported by an extensive quantity of known-origin and satellite tracked individuals (N = 227) to establish regional baseline values, indicating that the power of soft-tissue analyses may increase as baseline data becomes more readily available. We expect that the resolution achievable using barnacle isotopes will similarly improve as the technique is adopted more widely, with increased data availability allowing for more robust analyses.

The resolution of this method is dependent on the magnitude and/or rate of change in salinity and temperature, which drive the changes in the oxygen isotope values in barnacle shell^29,32^. Thus, it follows the resolution, and applicability, of this method may therefore be reduced in regions where sea temperature and/or salinity differences are less pronounced. However, the opposite is also true, with the resolution of this method likely improved in some other regions of the world.

We can make predictions about the expected resolution of this method in other regions of the world from existing δ^18^O_seawater_ data, a proxy for δ^18^O values in barnacle shell, that is influenced by salinity^29,49^. Across the geographic range of our study (eastern Queensland, Australia), LeGrande and Schmidt^50^ report that variation in δ^18^O_seawater_ may be as low as 0.2 to 0.4‰, whereas much larger changes (more than five times larger in some cases) are expected across similar distances in other regions (e.g. parts of the east coasts of both North and South America). This implies that at least some other regions are likely to provide higher resolution for identifying the origin of sea turtles from barnacle isotopes than we have presented here.

Future research would do well to address limitations in the understanding of spatio-temporal variation in δ^18^O_seawater_ and its relationship with salinity within each region for which these techniques are to be applied. This variable is required to convert between barnacle isotopes and water temperatures using the balanomorph barnacle paleotemperature equation^29^, but current knowledge comes from global databases of δ^18^O_seawater_ values measured across years (e.g.^50,51^), with no temporal variation and particularly sparse observations in the region of our study (the southwest Pacific). Given a relationship exists between salinity and δ^18^O_seawater_^49^, it is expected that regional values will vary with freshwater input.

Establishing robust regional equations of this nature (i.e. those that describe the relationships between SST, salinity, and barnacle isotopes) may allow isoscapes to be created from freely accessible, remotely collected data, and reduce the need for extensive in-water capture or satellite telemetry to support this method, perhaps eliminating this need altogether. In effect, this could allow large numbers of nesting turtles to be assigned foraging areas using a single barnacle sample from each individual, equating to identification of foraging area at approximately 1% of the cost of satellite telemetry, though at coarser scales. Analysing additional samples per turtle may further improve the resolution. Thus, despite the coarser resolution (compared with that provided by satellite telemetry), the method we present offers an opportunity to assess the foraging distribution of nesting sea turtle populations with higher replication (in a direct cost comparison) than other available methods (especially when considering soft-tissue isotope analyses require concurrent satellite telemetry), and at scales that remain useful to managers.

This study is the first to present a method that is able to distinguish between home foraging areas of sea turtles via SIA of the calcareous shells of commensal barnacles. This novel approach that combines barnacle growth rates to assign a date to each sample and stable isotope ratios from barnacle shell provides a basis for better understanding the foraging distribution of sea turtles. It is demonstrably possible to distinguish between barnacles formed in estuarine versus marine waters. It is also possible to separate areas spatially within marine waters by comparing isotopes, though possibly only at coarse spatial scales (i.e. 1,600 km as demonstrated here). It is possible to separate areas as little as 400 km apart with high success, but introducing areas in between can confound results and impede accuracy. This method could be used across other taxa and objects that carry barnacles, and further validation of methods may be able to improve the resolution at which areas can be separated.

## Methods

**Figure 2:**
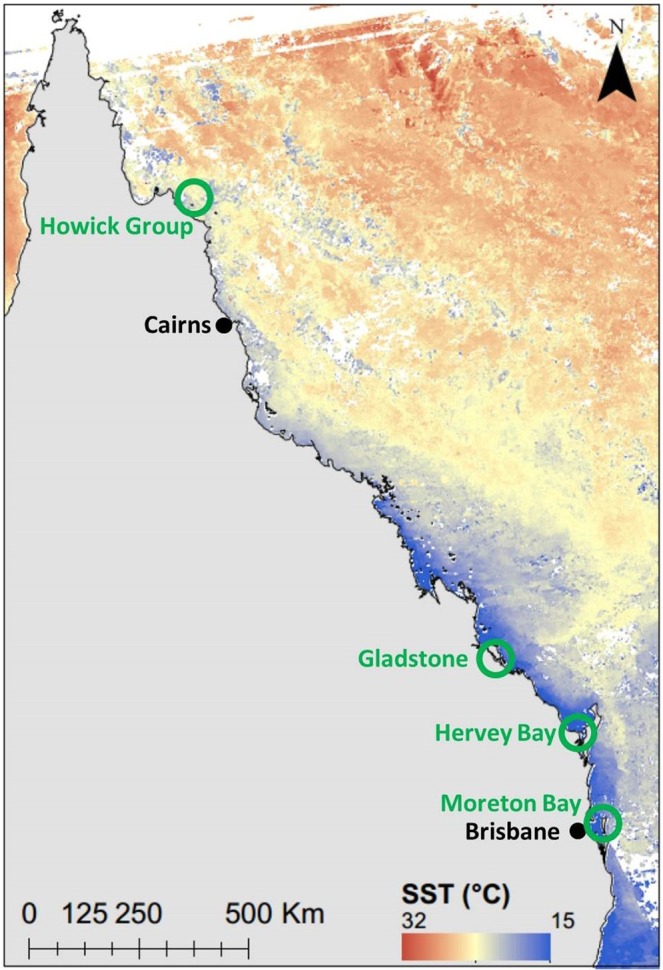
Map of Queensland, Australia, showing the four turtle foraging areas (green). Sea surface temperature (SST) at 15-Jun-2015. Data was sourced from the Integrated Marine Observing System (IMOS) – IMOS is a national collaborative research infrastructure, supported by Australian Government^62^.

Barnacles were collected from foraging and basking green and loggerhead turtles that were captured in four locations along the Queensland coast, spanning an area of approximately 1,600 km between July and October 2015. From north to south these foraging areas were the Howick group of islands, Gladstone, Hervey Bay, and Moreton Bay (Fig. 2).

The three southern areas are all coastal bays, partially enclosed by islands, and are generally described as estuarine environments^52,53^. However, the freshwater influence varies spatially and temporally within these systems^52–54^. As a result, we expect each to have experienced different freshwater influences during the sampling period.

The capture locations in Moreton Bay were all in the eastern side of the bay, an area that is likely to be influenced more by marine waters than by river discharges in the western bay. Salinity in the eastern bay is expected to remain above 34‰ except in cases of extreme flooding (e.g.^55^). Shimada, *et al*.^56^ demonstrated that turtles captured in the eastern bay tend to remain exclusively in this area or in the marine waters outside the bay (to the north and east) when not undergoing a breeding migration. This suggests a low likelihood that the Moreton Bay turtles captured in the present study would have encountered truly estuarine waters. Hervey Bay is often fully marine or even slightly hypersaline (>36‰) but salinity reduces to below oceanic levels following heavy rain in the catchment^57^. Our capture location in the western bay is approximately 6 km to the north of the Mary River mouth which, in combination with moderate rainfall over the period (140–170 mm May-July), suggests that the barnacles tested were likely exposed to lower salinities at some point in their growth. Similarly, parts of Gladstone Harbour are fully marine, but with rainfall triggering reductions in salinity at times^52^. Thus, we expect that our Moreton Bay samples will reflect a marine system, while Gladstone and Hervey Bay samples may return isotope ratios reflective of the variable freshwater influence expected in these locations. The northern area (Howick group) is expected to have experienced entirely marine conditions given that capture areas were in the coral lagoons of the northern Great Barrier Reef, 15–30 km from the mainland.

### Field sampling

Turtles were captured using the standard rodeo jump method^58^ in three of the four locations (Howick group, Gladstone, Moreton Bay). In Hervey Bay, turtles were instead encountered in mangrove flats while basking during the night time low tide. The largest available barnacle (*C. testudinaria*) was removed from the carapace or head of each turtle and dried to constant mass at 60 °C. All turtle capture and barnacle collection occurred between July and October 2015. Turtles were individually identified using titanium tags and key morphometric and demographic data were recorded as part of a long-running monitoring program.

This study was carried out in accordance with experimental protocols approved by both the Queensland Department of Agriculture, Fisheries and Forestry (DAFF) and Griffith University Animal Ethics Committees.

### Lab sampling

**Figure 3:**
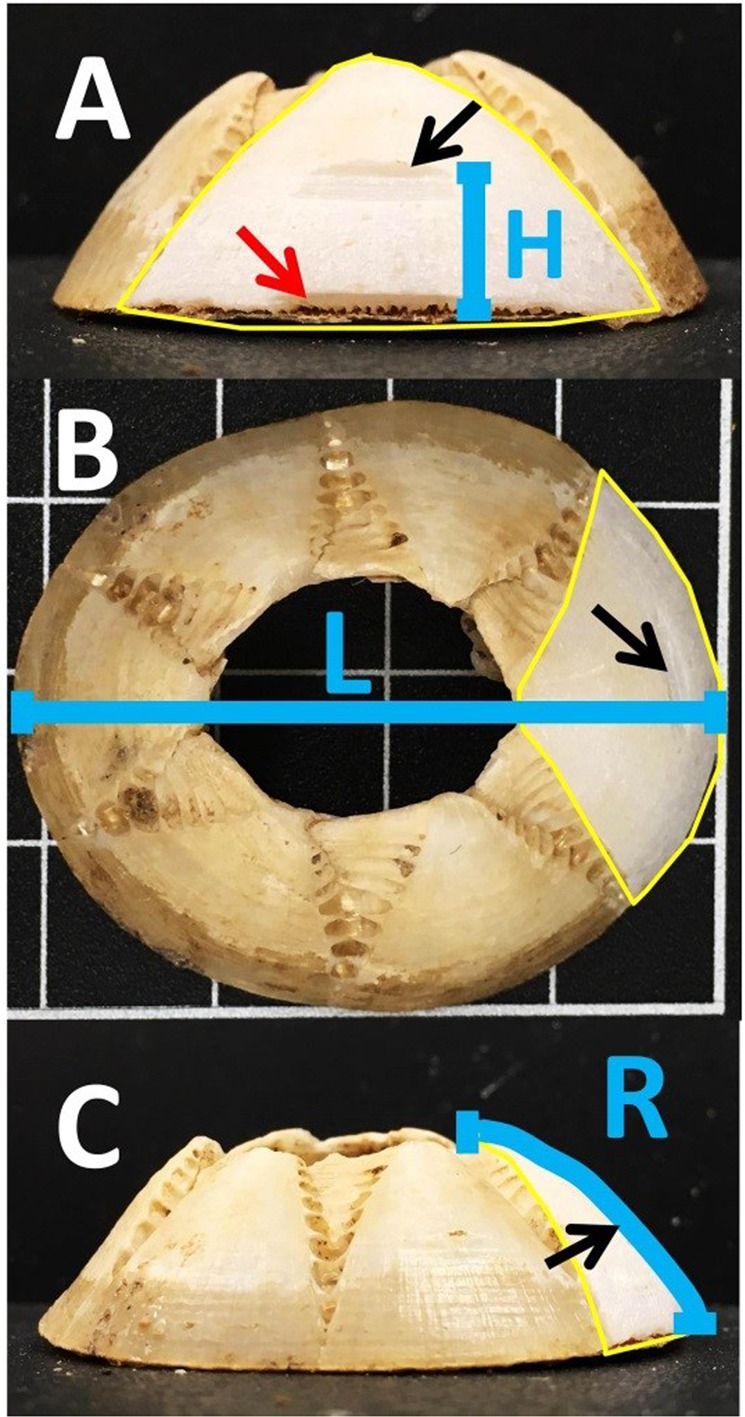
Summary of measurements used to calculate the age of each sample collected from a barnacle shell. (**A**) front view of barnacle facing rostrum. (**B**) top view of barnacle with rostrum on left. (**C**) side view with rostrum on right. Distance (mm) from base to centre of sample site (H); Maximum rostro-carinal length in mm (L); Length (mm) of curved surface of rostrum (R). Red arrow points to sample taken of newest/youngest material. Black arrow points to the oldest of three successive samples taken from this barnacle. Yellow line identifies the extent of the rostrum plate in each frame. Each grid-square (in **B**) is 10 mm.

After drying, the maximum rostro-carinal length (Fig. 3B) and curved length of the rostrum surface (Fig. 3C) was recorded for each barnacle. These variables are used in age calculation and for assigning dates to sub-samples based on the growth curve described by^44^. Only barnacles without obvious shell deformities were used in order to maintain confidence in sample age assignments. Deformities include conspicuous curvature or indents to the basal disk or shell plates. These deformities affect shell formation and thus complicate age estimates. The basal disk of each barnacle was coated with epoxy resin to minimise breakage and contamination during the sample extraction process. Each barnacle was cleaned mechanically using a hand-held Dremel rotary tool, removing all epiphytic growth, dirt and discolouration. Care was taken to ensure minimal calcite was removed from the outer layer. Remaining dust was mechanically removed using a Dremel with brush tip and a manual air pump.

For balanomorph barnacles, the youngest shell material is near the outer edge of the base (e.g. the bottom of frames A & C in Fig. 3), with layers becoming progressively older with increasing height along the shell surface^33^. Therefore, target areas were described as a distance (in mm) along the rostrum from the base.

Each barnacle was sampled multiple times along the outer edge of the rostrum where shell material was calculated to have formed between May and July 2015. Each sample was from a different location and thus represented a different time within this period. We set the target date as the 15^th^ of each month and calculated the location (as distance from base – Fig. 3A) where shell material of this age was formed for each barnacle using the von Bertalanffy growth equation for *Chelonibia testudinaria* described by^44^. This area was then sampled as described below. Post-sample extraction measurements were taken from the base to the centre of the sample site (distance from base, in mm). The age was then calculated, and each sample was assigned an individual formation date.

The same dates were targeted between foraging areas because it was not possible to compare isotope values directly to environmental parameters in this case. High-resolution data (temporally and spatially) for temperature and salinity were not available for the time period and locations we assessed. Some coarse temperature data were available, but salinity data were not, and approximating from historical mean data does not suitably account for variability within the periods and locations represented in each individual sample. Thus, by identifying differences between isotope values of shell material formed at the same time but in different areas, we are able to infer that temperature and salinity was different between areas due to the known effect of these parameters on barnacle shell isotopes^29,32^.

For all subsequent statistical analyses, samples were categorised by the month in which they were expected to have formed. Equations for calculating the date represented by a sample are presented in the On-line Supplement (Appendix S1: Eqs S1–S6). Some samples were unusable for various reasons (e.g. the median date calculated post-extraction fell outside the target months; insufficient sample mass collected). These samples were not analysed and prevented further sampling at or near the target location, resulting in an unbalanced design (i.e. different numbers of samples from each barnacle).

Green and loggerhead turtles in the western Pacific are known to show high fidelity to foraging areas, using the same foraging areas pre- and post-migration, and across years^4^. There is no evidence that these turtles use different foraging areas seasonally, unlike those in some other regions^4^. Migrations generally occur between October and March, with only adult turtles undertaking migrations during these months. Thus, we expect all turtles to have remained in the area near to where they were captured for the full period represented by our samples.

Extraction of shell samples was performed using diamond coated cutting blades (0.6 mm thickness) in a Proxxon MF-70 micromill with rotating dividing head. The blade was aligned with the target area and inserted 0.8 mm after first contact. The barnacle was then rotated on the dividing head approximately 5° along the growth axis (parallel to base) to capture enough material of the same age for analysis. Calcite powder was captured in a 1.5 mL microcentrifuge tube and then analysed for stable carbon (δ^13^C) and stable oxygen (δ^18^O) isotope ratios.

### Methodological reproducibility

Additional samples were used to test variation within a barnacle for samples of the same age. We extracted adjacent samples separately from the same barnacle (where sample age calculations were within two days of one another), analysed for δ^18^O and δ^13^C ratios, and calculated differences between paired samples (N = 20 pairs. One pair from each of 20 different barnacles).

### Isotope analysis

Analysis of δ^13^C and δ^18^O in barnacle calcite was performed at the Australian National University Research School of Earth Sciences Stable Isotope Laboratory (Canberra, Australia) using Finnigan MAT 251 and 253 mass spectrometers fitted with automated individual-carbonate reaction (Kiel) devices. Where sample weights were too low for the MAT 251 (<100 µg), the MAT 253 was used. CO_2_ was liberated from the carbonate by reaction under vacuum with 105% H_3_PO_4_ at 90 °C (MAT 251) and 75 °C (MAT 253). In both cases, measurements of δ^18^O and δ^13^C were corrected using the NBS19 (δ^18^O = −2.20‰, δ^13^C = 1.95‰) and NBS18 (δ^18^O = −23.0‰, δ^13^C = 5.0‰) standards and are reported in delta notation relative to Vienna Peedee Belemnite (VPDB). Analytical precision for repeat measurements of NBS-19 run in parallel with the shell samples was 0.03‰ for δ^18^O and 0.02‰ for δ^13^C (1 S.D., N = 46).

### Data analyses

#### Testing for isotopic differences between foraging areas

Barnacle shell isotope ratios, from samples collected in each foraging area, were analysed using MANCOVA in SPSS^59^ to assess if the ratios were statistically different between areas. ‘Area’ was used as a factor and sample ‘month’ (i.e. the month in which the sample was expected to have been formed) was used as a covariate. Post-hoc least significant difference (LSD) tests identified statistical differences between paired areas.

#### Foraging area assignments

Linear Discriminant Analysis (LDA) MASS package in R v3.41^60,61^, was used to assign individual samples to foraging areas as in^26^, and to investigate the spatial scale at which this method may be useful in eastern Australia. For all tests, ‘area’ was set as the grouping variable with δ^18^O, δ^13^C, and month used as predicting variables. Priors were set so that there was an equal probability that a sample could come from all areas included, rather than being reflective of the sample proportions in the calibration dataset. For example, where four areas were included, the probability of coming from any individual area was set to 0.25. In this analysis, the calibration dataset defines which values of predicting variables best describe each level in the grouping variable. These characteristics are then used to assign individual samples in the validation dataset to one of the levels in the grouping variable. Assignment success is measured as a percentage based on the number of correct assignments within a level and overall.

There are many ways that calibration and validation datasets could be defined. Therefore, in order to establish if there was any bias on assignment success developing from how datasets were separated, we tested multiple methods for defining which samples were used in each. The full dataset (sample N = 93, turtle/barnacle N = 27) was split into two subsets (one for calibration, one for validation of the model) using multiple methods across 12 iterations (2 sample splitting techniques × 3 methods for defining areas × 2 directions, forward and reverse).

For sample splitting techniques, first, we split by alternating assignment of individual samples (Alternating Samples; AS). This method was designed to maintain the highest possible replication at the turtle level (turtle N between 21 and 26 within each of the two datasets across tests), while ensuring that no individual sample was represented in both datasets. A limitation of this method is that the same turtle was often represented in both datasets, but by different samples from different deposition dates. The second sample splitting method was by alternating turtles, where all samples from individual turtles were assigned to a single dataset (Alternating Turtles; AT). This ensured independence of samples and turtles between datasets, but limited replication at the turtle level (turtle N between 11 and 14 within each dataset across tests).

To test the spatial scale at which this method may be useful for assigning foraging areas to nesting turtles in eastern Australia, we ran the analysis (using the same splitting methods) with three methods for defining areas: (1) all four areas included (4 areas), (2) Hervey Bay excluded (3 areas), and (3) with all southern areas grouped together (South) and Howick samples used as a North group (2 areas). Hervey Bay was chosen for exclusion in the second test because it was an intermediate location, between Gladstone and Moreton Bay, thus changing the minimum distance between areas from approx. 150 km to 400 km and enabling assessment of effective spatial resolution. Similarly, to test the effect of expanding the minimum distance between areas dramatically, we grouped the three southern areas and re-ran the analysis with only two groups (North and South). These had a minimum distance between them of approximately 1,100 km.

## Supplementary information

Supplementary Information

## Data Availability

The datasets generated during and/or analysed during the current study are available from the corresponding author on reasonable request.
