# Supplementary material for: Distinguishing between sea turtle foraging areas using stable isotopes from commensal barnacle shells
Source: Sci Rep. 2019 Apr 25;9:6565. doi: 10.1038/s41598-019-42983-4 (PMC6483986; doi:10.1038/s41598-019-42983-4)
Supplement: Supplementary file 1 — Supplementary Information [file 41598_2019_42983_MOESM1_ESM.docx]

**Appendix S1. Online Supplement for** “*Distinguishing between sea turtle foraging areas using stable isotopes from commensal barnacle shells*”

Ryan M. Pearson*^1^, Jason P. van de Merwe^1^, Michael K. Gagan^2^, Colin J. Limpus^3^, Rod M. Connolly^1^

**Equations for calculating barnacle age and sample age**

**Equation S1. von Bertalanffy growth equation for *Chelonibia testudinaria* presented by Doell *et al.* (2017)**

$$L=69.26\left( 1-0.9907e^{-0.003678t} \right)$$

**Equation S2. Rostrum:Length ratio**

$$R:L= \frac{Rostrum}{Length}$$

**Equation S3. Length at sample**

$$Length at sample (Ls)= \frac{Rostrum length-distance from base}{R:L}$$

**Equation S4. Barnacle age at capture (in days) from length; Note: substituting length (L) with calculated length at sample (Ls) gives ‘Age at sample’.**

$$Age \left( days \right)= \frac{ln(\frac{-69.26*0.9907}{L-69.26})}{0.003678}$$

**Equation S5. Sample days before capture**

$$Sample days before capture=(age at capture)-(age at sample)$$

**Equation S6. Date at sample (median)**

$$Date=Capture date-Age at sample$$

**Example S1. Worked example:**

**Capture date:** 11-Jul-2015; **Length:** 32.72 mm;

**Rostrum:** 12.0 mm; **Sample distance from base:** 2.51 mm

1. **R:L** = 12.0/32.72 = 0.36675
2. **Ls** = (12.0 – 2.51)/0.36675 = 25.876
3. **Age at capture** = ln((-69.26*0.9907)/(32.72-69.26))/0.003678 = 171.3 days
4. **Age at sample** = ln((-69.26*0.9907)/(25.876-69.26))/0.003678 = 124.7 days
5. **Sample days before capture** = 171.3 – 124.7 = 46.7 days
6. **Date at sample** = 11/Jul/2015 – 47 days = 25/May/2015

Table S1. Results of Linear Discriminant Analyses (reverse iterations only) using multiple methods to define calibration and validation datasets. Split method defines how each sample was selected for the calibration or validation datasets. Areas: the number of regions which were used as the grouping variable. Cal: The number of turtles and samples used in the calibration subset and; Val: in the validation subset. Assignment success per area is defined as the number of samples (S) and turtles (T) that were correctly assigned to the areas they were from. Overall Accuracy: the percentage of samples (S) and turtles (T) correctly assigned to their home area across all areas used. Mean Accuracy: the average percentage of samples (S) and turtles (T) that were assigned correctly across both directions. South & North groupings used where only two areas are included. Gladstone, Moreton, and Howick used in three area tests.

| **Split method** | **Areas** | **Sample Sizes (N)** | | | | | | **Assignment success per area (%)** | | | | | | | | | | **Overall Accuracy (%)** | | **Mean accuracy (%)** | |
| --- | --- | --- | --- | --- | --- | --- | --- | --- | --- | --- | --- | --- | --- | --- | --- | --- | --- | --- | --- | --- | --- |
|  |  | **Turtles** | | | **Samples** | | | **South** | | | | | | | | **North** | |  |  |  |  |
|  |  |  |  |  |  |  |  | **Gladstone** | | | **Hervey** | | | **Moreton** | | **Howick** | |  |  |  |  |
|  |  | **Total** | **Cal** | **Val** | **Total** | **Cal** | **Val** | **S** | **T** | | **S** | **T** | | **S** | **T** | **S** | **T** | **S** | **T** | **S** | **T** |
| **Alternating samples (AS)** | **4** | 27 | 26 | 25 | 93 | 46 | 47 | 50 | 50 | | 67 | 75 | | 71 | 80 | 100 | 100 | 72 | 76 | **71** | **78** |
|  | **3** | 23 | 22 | 21 | 70 | 35 | 35 | 63 | 67 | | NA | | | 92 | 89 | 93 | 100 | 83 | 90 | **85** | **86** |
|  | **2** | 27 | 26 | 25 | 93 | 46 | 47 | **S** = 94; **T** = 92 | | | | | | | | 100 | 100 | 97 | 92 | **97** | **94** |
| **Alternating turtles (AT)** | **4** | 27 | 13 | 14 | 93 | 49 | 44 | 78 | 75 | 0 | | 0 | 42 | | 40 | 100 | 100 | 55 | 57 | **53** | **59** |
|  | **3** | 27 | 11 | 12 | 70 | 37 | 33 | 100 | 100 | NA | | | 83 | | 80 | 100 | 100 | 94 | 92 | **88** | **91** |
|  | **2** | 23 | 13 | 14 | 93 | 49 | 44 | **S** = 94; **T** = 91 | | | | | | | | 100 | 100 | 97 | 93 | **97** | **93** |

**References**

Doell, S.A., Connolly, R.M., Limpus, C.J., Pearson, R.M. & van de Merwe, J.P. (2017) Using growth rates to estimate age of the sea turtle barnacle *Chelonibia testudinaria*. *Marine Biology,* **164,** 222.
